# Healthcare Human Resources: Trends and Demand in Saudi Arabia

**DOI:** 10.3390/healthcare9080955

**Published:** 2021-07-29

**Authors:** Khalid Alnowibet, Adel Abduljabbar, Shafiq Ahmad, Latifah Alqasem, Nabil Alrajeh, Luigi Guiso, Mazin Zaindin, Madhusudhan Varanasi

**Affiliations:** 1 Department of Statistics and Operations Research, College of Science, King Saud University, Riyadh 11451, Saudi Arabia; lfalqasem@gmail.com (L.A.); zaindin@ksu.edu.sa (M.Z.); 2 Department of Psychology, College of Education, King Saud University, Riyadh 11451, Saudi Arabia; abduljabbar@ksu.edu.sa; 3 Department of Industrial Engineering, College of Engineering, King Saud University, Riyadh 11451, Saudi Arabia; ashafiq@ksu.edu.sa; 4 Department of Biomedical Technology, College of Applied Medical Sciences, King Saud University, Riyadh 11451, Saudi Arabia; nabil@ksu.edu.sa; 5 Department of Economics, Institute for Economics and Finance, 00118 Rome, Italy; luigi.guiso@tin.it; 6 Department of Management, College of Business Administration, Al-Yamamah University, Riyadh 11451, Saudi Arabia; v_prasad@yu.edu.sa

**Keywords:** saudization, healthcare human resources planning, private sector hospitals, saudi vision 2030, key performance indicators

## Abstract

This paper estimates the impact of policies on the current status of Healthcare Human Resources (HHR) in Saudi Arabia and explores the initiatives that will be adopted to achieve Saudi Vision 2030. Retrospective time-series data from the Ministry of Health (MOH) and statistical yearbooks between 2003 and 2015 are analyzed to identify the impact of these policies on the health sector and the number of Saudi and non-Saudi physicians, nurses and allied health specialists employed by MOH, Other Government Hospitals (OGH) and Private Sector Hospitals (PSH). Moreover, multiple regressions are performed with respect to project data until 2030 and meaningful inferences are drawn. As a local supply of professional medical falls short of demand, either policy to foster an increase in supply are adopted or the Saudization policies must be relaxed. The discrepancies are identified in terms of a high rate of non-compliance of Saudization in the private sector and this is being countered with alternative measures which are discussed in this paper. The study also analyzed the drivers of HHR demand, supply and discussed the research implications on policy and society. The findings suggest that the 2011 national Saudization policy yielded the desired results mostly regarding allied health specialists and nurses. This study will enable decision-makers in the healthcare sector to measure the effectiveness of the new policies and, hence, whether to continue in implementing them or to revise them.

## 1. Introduction

The declining oil prices and increasing global focus on renewable energy are compelling reasons for the Saudi Government to consider non-oil sectors for attaining sustainable economic growth and to maintain and improve the welfare of the nation in general and with respect to healthcare in particular. The healthcare system in Saudi Arabia is divided into three sectors: The Ministry of Health (MOH) sector, the Other Government Healthcare (OGH) sector and the Private Healthcare Sector (PHS). According to the health rules and regulations, the MOH has the responsibility of providing healthcare services in hospitals and secondary and specialized treatment centers as well as developing the health strategy and plans for the provision of healthcare in the Kingdom. Therefore, all three segments of the healthcare system are legislated by the MOH. While healthcare in the Kingdom is provided by both public and private sectors, the majority of healthcare services (60%) is provided by the MOH sector, while the OGH and PHS contribute 20% each [1]. Between 2003 and 2015, government spending on healthcare remained between 6.49% and 7.25% of the total government budget [2]. The MOH reported that more than 75% of the total health expenditure in the Kingdom is from government funding [2]; public money is, thus, the primary funding source.

### 1.1. Healthcare Policies of Saudi Arabia

Saudi Vision 2030 identified greater opportunities for the private sector to operate in core sectors, such as healthcare, utilities, industry and Information and Communication Technologies (ICT) [3]. While public health organizations in the Kingdom continue to be critical, there is a need to shift to private healthcare sector for the long term. Saudi Vision 2030 also envisages the need to reduce the unemployment rate from 11.6% in 2016 to 7% by 2030 and to raise women’s employment rate 30% by 2030 from 22% in 2016. The health sector is considered to provide good employment opportunities for Saudi nationals [1].

The National Transformation Program 2020 envisaged several strategic objectives and key performance indicators (KPIs) for the health sector to be increased from 2015 (baseline) to 2020 (target). For instance, the level of private area commitment in absolute medical services is expected to increase from 25% to 35%; the number of occupant Saudi doctors who are taken on in preparing projects is to be expanded from 2200 to 4000; the number of qualified Saudis in the field of nursing and care staff for each 100,000 individuals is to be expanded from 70.2 to 150; the level of patients who had crisis or pressing consideration with clinical choices made (affirmation/move/release) in under 4 h in key clinics is to be expanded from 40% to 75%; the number of essential medical service visits per capita is to be expanded from 2 to 4; the number of authorized clinical offices (subsidiary with the MOH and private) is to be expanded from 40% to 100%; the percentage of arrangements obtained from specific clinical orders within a month (normal for all fortes in key emergency clinics) is to be expanded from under 40% to 70%; the percentage of medical care offices announcing thorough execution and quality measures is to be expanded from 10% to 100%; all out income produced from private areas from using government wellbeing assets is to be expanded from 0.3 SAR billion to 4 SAR billion; the level of patients who receive medical services after basic consideration and long haul hospitalization inside about a month is to be expanded from 25% to half; the level of medical clinics that meet the US middle for patient security culture is to be expanded from 10% to half [1].

### 1.2. Research Problem

Healthcare is the priority of any nation and quality of service cannot be compromised at any cost. Globally, the health sector is attracting more women employees who can manage both job and family responsibilities [4]. Culturally in Saudi Arabia, the majority of men believe that women performing nursing jobs reduce their social status. A State-driven campaign to change Saudi men and women’s adverse beliefs towards nursing jobs may contribute to motivating more women to work in the health sector with dignity and this will expand the supply of local labor. This, however, may take time. Currently, due to the resistance of Saudi women to engage in the nursing profession and considering their lower cost with same or better quality, expatriates may be encouraged to fill the shortages in the short term and, if necessary, in the long term. Physician workforce planning should include an understanding of the issues and policy strategies that can affect the workforce development and be integrated with planning of medical education, health services and financing health human resources [5,6,7]. The feminization of the medical workforce has been a major change over the past 20 years [6,8].

The Saudi Government developed a series of laws and policies for the growth of the economy and employment of Saudi nationals. The Saudi Government introduced a law in 1999 [9] requiring the private sector to provide insurance to expatriates, which enhanced the growth of the private health insurance sector and the subsequent growth of private hospitals. In 1999, doctors in government hospitals had the opportunity to enhance their income through a dual career in private hospitals. In 2007, in order to improve the quality of services in government hospitals, the government prohibited the concept of a dual career. In addition, the 2011 Nitaqat program [10], which compels private sector companies to appoint local residents, has increased the burden of non-availability of dedicated and specialized doctors and nurses.

### 1.3. Objectives

The goal of this research paper is to study the impact of governmental policies on the healthcare human resources (HHR) inventory in Saudi Arabia in terms of trends in demand supply as well as the enhancement of Saudi nationals’ participation in the healthcare sector. The objectives of this paper are the following:

- To conduct a time-series analysis of national and international health human resources data in order to determine their percentage change in MOH, OGH and PHS;
- To extrapolate the growth of the population of Saudi Arabia until 2030 and, accordingly, project the demand for HHR from 2018 until 2030;
- To understand the drivers of HHR demand and supply in 2015, 2020 and 2030;
- To analyze the results in terms of the impact of Government policies on attracting Saudi nationals to the healthcare sector;
- To offer suggestions in terms of bridging the gap between theory and practice and the impacts on policies and the society.

## 2. Methodology

### 2.1. Data Collection

The data used in this analysis are aggregated data collected from statistical reports published by the MOH. The MOH statistical reports are issued annually and it addresses several aspects of the health sector in Saudi Arabia. At the time of this research, the statistical reports were available only during the period between 2003 and 2015. The HHR data are extracted from a total of more than 65 [2] tables available online. The extraction process was performed in several stages.

### 2.2. Data Transformation

First, the available statistical reports were non-editable and in different formats for different years. Therefore, the tables were transformed into Microsoft Excel format using the “Able2Extract Professional 11.0” software. Secondly, some of the reports published data as images, which requires keying in the data manually. Later, it was discovered that the data of interest were not presented in a standard format because of the administrative transitions that MOH experienced during these years. Thus, the data tables were restructured to all be in a similar format. Finally, the collected data were combined as data series in three main healthcare sectors: MOH, OGH and PHS. Each sector is divided into three categories of HR: physicians, nurses and allied health specialists. In addition, each category is divided into two sub-categories based on nationality (national/international).

We identified Saudi Arabian policy documents relating to HR in healthcare from the MOH website and by conducting a review of published articles concerning medical policies in the Kingdom. We searched for documents that were published by the Saudi Arabian Government health department related to the 2020 national transformational plan (NTP) and the 2030 Vision.

### 2.3. Statistical Tools and Techniques

The quantitative data analysis included time series analysis, univariate analysis and multiple regressions using SPSS 23.0. We generated knowledge on HRH in the Kingdom by using data from twelve years of reported data on the healthcare sector of Saudi Arabia. Data are analyzed and regressed overtime for three separate sectors: (1) MOH, (2) OGH and (3) PHS. In order to draw meaningful inferences, the results are analyzed while considering various Saudi Arabian healthcare sector policies.

## 3. Results and Discussion

The analysis is based on secondary data collected by the MOH, OGH and PHS in Saudi Arabia. The discussion is based on the data analysis of the employment of physicians, nurses and allied health personnel by the MOH, OGH and PHS in the Kingdom.

### 3.1. Ministry of Health (MOH) Sector

**Figure 1:**
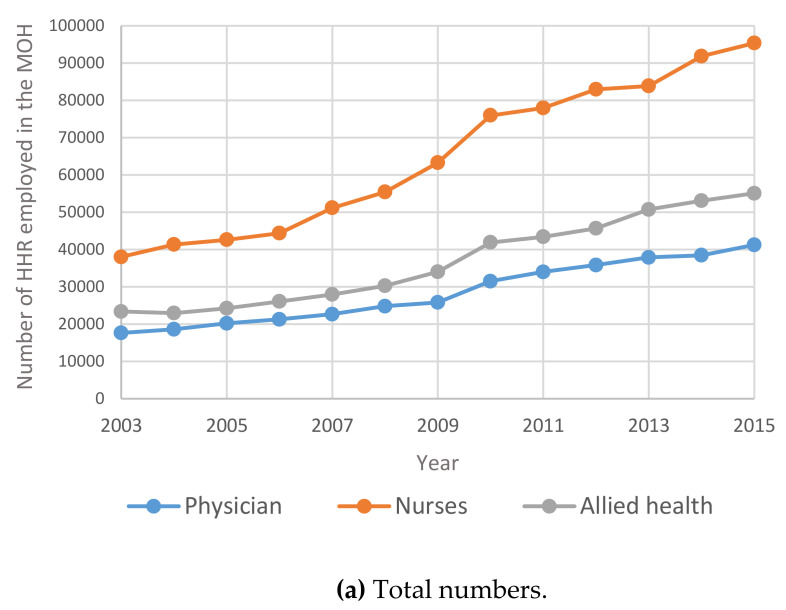

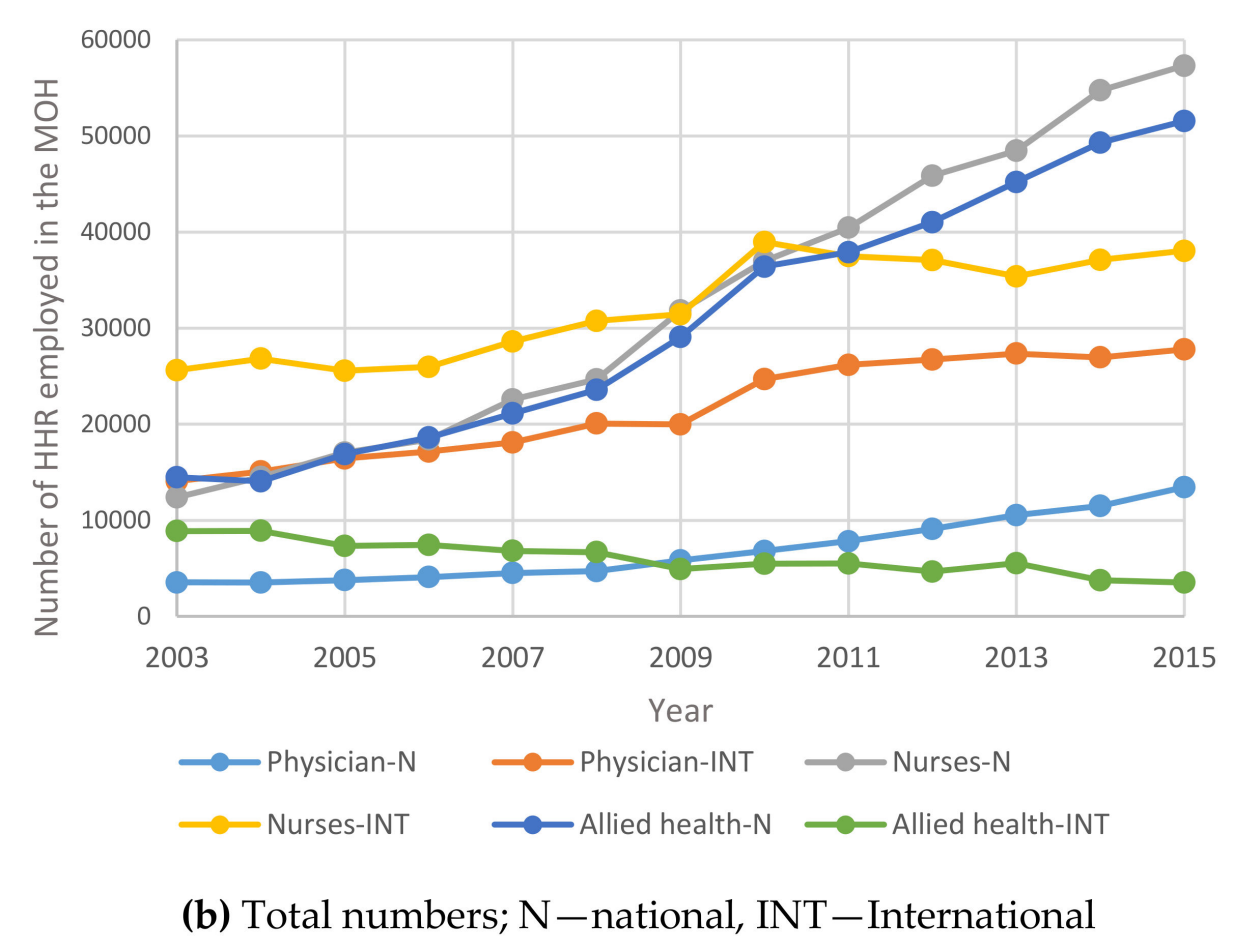
Time Series for HHR in the MOH between 2003 and 2015: (**a**) total numbers; (**b**) total numbers; N—national; INT—International.

Figure 1a illustrates the time series of the total number of doctors, nurses and allied health human resources in the MOH. Figure 1b documents trends in the numbers of doctors, nurses and allied health human resources separately for Saudi nationals and international human resources during the period between 2003 and 2015.

**Figure 2:**
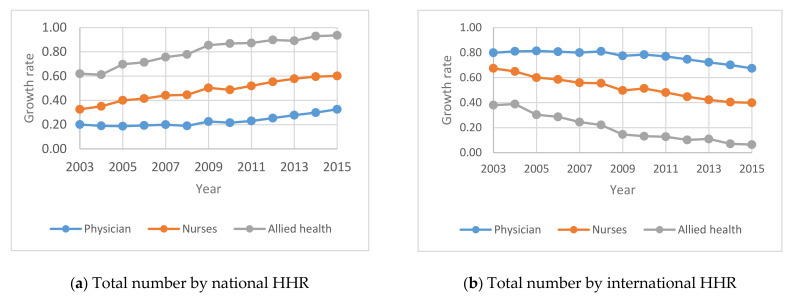
Annual Percentage change of national and international HHR in the MOH between 2003 and 2015: (**a**) total number by national HHR; (**b**) total number by international HHR.

Figure 2 shows the evolution of the composition of HHR between 2003 and 2015 for both Saudi and non-Saudi personnel in each category within the MOH.

From Figure 1 below, it may be noted that the total number of HHR in Saudi Arabia increased year on year from 2003 to 2015. It is interesting to note that due to the Nitaqat program in 2011, there is a steady increase in the number of Saudi physicians, nurses and allied health specialists in MOH and a proportionate decrease in the respective international HHR in Saudi Arabia from 2011 onwards.

Figure 2 indicates that the percentage change in the Saudi national HHR in the MOH increased between 2011 and 2015 and correspondingly decreased between 2011 and 2015 for international HHR in Saudi Arabia. This may be attributed to the policy changes such as Nitaqat 2011 to usher the employment of Saudis in the MOH sector.

### 3.2. Other Government Healthcare (OGH) Sector

**Figure 3:**
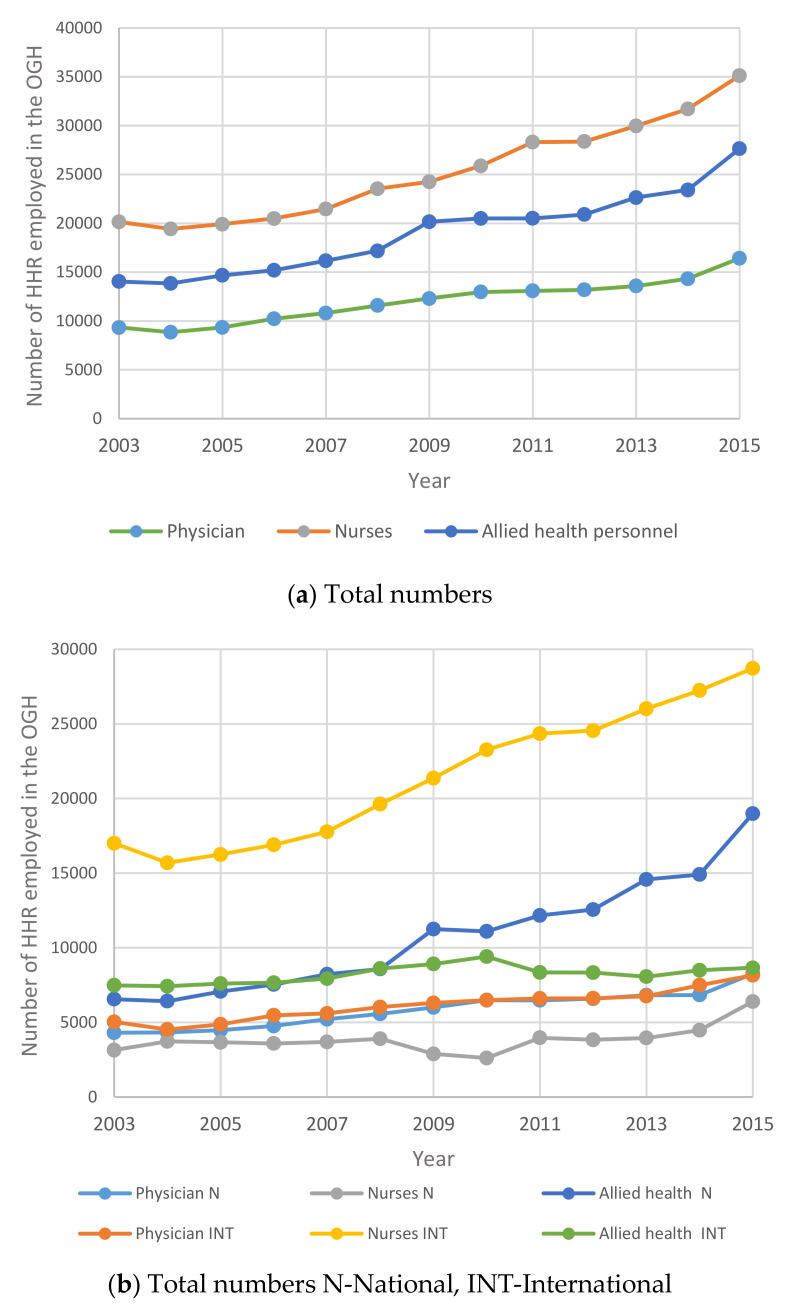
Time Series for HHR in OGH between 2003 and 2015: (**a**) total numbers; (**b**) total numbers N—National; INT—International.

**Figure 4:**
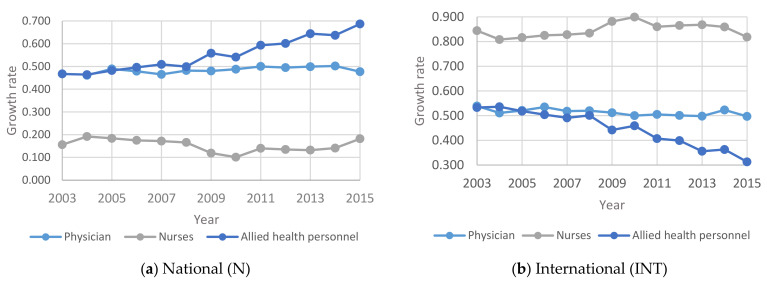
Annual Percentage changes for HHR in the OGH sector between 2003 and 2015: (**a**) National (N); (**b**) International (INT).

In this section, the focus is on studying human resources in the other government healthcare sector. Figure 3 shows the growth rate of human resources in the three primary categories of physicians, nurses and allied health personnel operating in the other government healthcare (OGH) sector in the Kingdom. Figure 4 shows the annual Percentage changes for HHR in the OGH sector between 2003 and 2015.

Figure 3 above indicates that the total number of HHR in Other Government Hospitals in Saudi Arabia increased steadily from 2003 to 2015. The number of Saudi nurses and allied health specialists outnumbered the international nurses and all health specialists. It is interesting to note that the number of Saudi physicians is almost equal to the number of non-Saudi (expatriate) physicians in the OGH.

According to Figure 4 the percentage of total national HHR in the OHG increased from 2011 to 2015. While the percentage of international nurses and allied health specialists decreased over some time, the graph of the percentage of international physicians is almost flat from 2009 to 2015. This can be attributed to the government initiatives to increase the number of Saudi nationals in the employment market in general and the health sector in particular.

### 3.3. Private Healthcare Sector (PHS)

**Figure 5:**
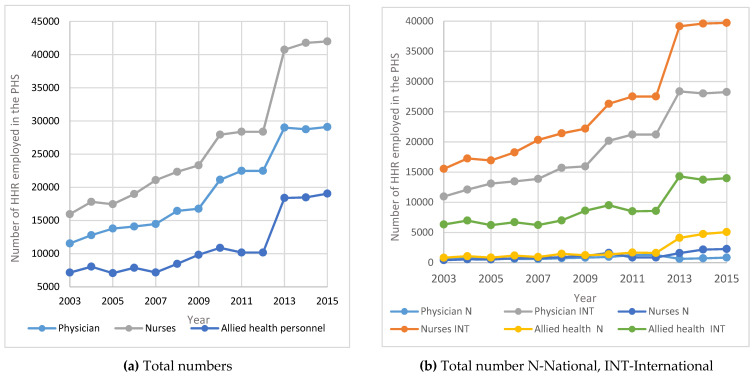
Time Series for HHR in the PHS between 2003 and 2015: (**a**) Total numbers; (**b**) total number N—National; INT—International.

**Figure 6:**
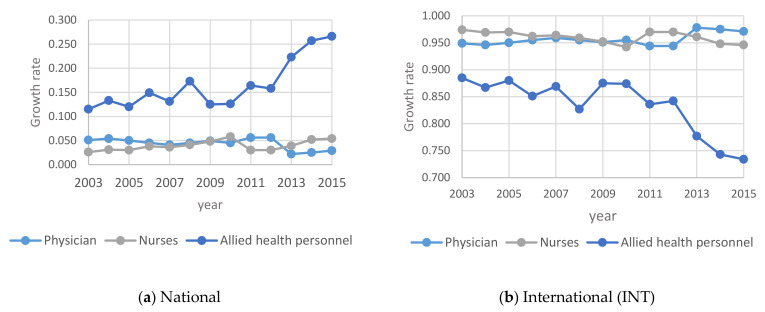
Annual Percentage change for HHR in the PHS between 2003 and 2015: (**a**) National; (**b**) International (INT).

In the Kingdom of Saudi Arabia, there is a need for private hospitals to enhance healthcare services in order to meet the rising number of Saudi and non-Saudi patients. The private segment of the health system also contributes to the overall efficiency of the supply of health services as it places some competitive pressure on the public segment. Figure 5 shows the time Series for HHR in the PHS between 2003 and 2015. Figure 6 shows the annual Percentage change for HHR in the PHS between 2003 and 2015.

Figure 5 implies that for the PHS, the number of HHR increased dramatically between 2003 and 2015. This is because of the Saudization that was introduced in 2003 and the Nitaqat program in 2011. However, in terms of the absolute numbers, the expatriate health professionals outnumbered Saudis in the private healthcare sector.

However, from Figure 6 it may be noted that for the national HHR, the percentage change increased significantly in PHS between 2003 and 2015 and decreased accordingly for the international HHR during the same period. This means that more Saudis are employed in the place of the expatriates.

### 3.4. Projection of Population and Demand of HHR

**Figure 7:**
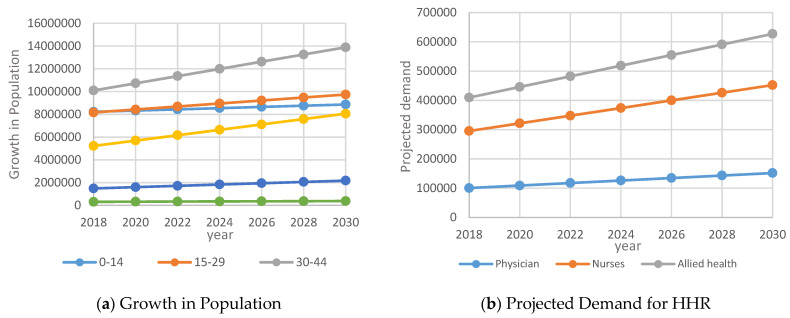
Extrapolated data for growth in population and HHR between 2018 and 2030: (**a**) Growth in Population; (**b**) Projected Demand for HHR.

Under the assumption that there will be significant changes economically and socially, the population will have the same growth rate until 2030. Therefore, it could be assumed that the projection of the population is a simple linear regression model. Figure 7a, a projection of the population’s ages is derived under; Figure 7b shows extrapolations of health care human resources demand over the 12 years from 2018 to 2030.

Figure 7a indicates that approximately 21% of the Saudi population is younger than 14 years of age, 54% of the Saudi population is between the ages of 15 and 44 and 16% are between the ages of 45 and 59. Hence, the Saudi Government is determined to develop their talents and to invest in their productive capabilities to support their contribution to the development of Saudi society and economy [1].

Figure 7b indicates the demand for physicians, nurses and allied health specialists between 2018 and 2030. Policies to enhance the capacity of healthcare-related educational institutions, planned high-quality education and incentives may motivate many Saudis to pursue a career in the health sector. In the event of a shortage, international physicians, nurses and allied specialists may be attracted. As the pressure on the PHS is increasing, there could be a shortage of HHR from within Saudi Arabia and, in particular, in terms of quality, quantity and cost-benefit to provide quality healthcare. Healthcare is an essential requirement for the growth and development of the nation and the Saudization policy may be relaxed for certain categories of hospitals on a case-by-case basis.

### 3.5. The Supply of Healthcare Human Resources

From Figure 7a, it may be noticed that the population of Saudi Arabia is likely to increase from 31.56 million in 2015 to 35.09 million by 2020 and 43.1 million by 2030. Accordingly, the demand for physicians is likely to increase from 86,756 in 2015 to 109,054 by 2020 and 153,462 by 2030. Similarly, the demand for nurses, pharmacists and allied health personnel is likely to increase as shown in Table 1.

Table 1 provides the breakup of demand for physicians, nurses, pharmacists and allied health personnel in MOH, Other Government Hospitals and private sector hospitals in Saudi Arabia by 2020 and 2030.

According to NTP2020, the number of qualified Saudis in the field of nursing and support staff for every 100,000 population is to be increased from 70.2 (baseline) to 150 by 2020. According to our estimation in Table 1, the number of nurses required for a population of 35.09 million is 224,278, which comes to be about 156 per 100,000 populations. If the demand for nurses mentioned in Table 1 is met, the NTP2020 Strategic Objective (which aims at increasing the attractiveness of nursing and medical support staff as a preferred career path) will be achieved.

The MOH has set the ball rolling to achieve the goal of employing 100,000 Saudis in the health care sector as part of the Vision 2030 plan. The ministry aims to nationalize health specialties with competitive and qualified staff. It will implement a performance measurement system and define specifications for health licenses as well as provide training opportunities by increasing the number of specialized seats. The ministry’s partnership with specialized educational and academic establishments is expected to help in achieving the 100,000 targets by the year 2030.

### 3.6. Drivers of HHR Demand

Table 2 indicate the demand for HHR in 2020 and 2030. The forecasting of demand for HHR is much more difficult than the supply because a greater number of potential factors may affect the future demand for health services and the uncertainties surrounding most of the factors such as projected growth in population size, health care needs by age, sex and epidemiological factors, future GDP growth and health care budget or expenditure growth on the demand for MOH, OGH and PHS.

As it can be observed from Table 2, it is evident that the supply of health human resources in the Kingdom of Saudi Arabia is far less than the demand. The spirit of Saudization is not only to reduce and end unemployment but also to compete internationally. While the Saudization goals are by and large achieved in the MOH and OGH, there are discrepancies in the policy. For example, the 2018 MOH statistics show that in the private sector, expatriate healthcare professionals comprise 90 percent out of the workforce of 124,000 that consists of doctors, nurses and pharmacists. Out of a total of 33,800 doctors in the private sector, there are only 3000 Saudi doctors which is about 8% of Saudization. Similarly, out of 45,900 nurses in the private sector only 2700 are Saudi nurses, i.e., about 6% Saudization. While there are 22,000 pharmacists in the private health sector only 1100 i.e., 5% are Saudis. Out of 22,000 medical assistants, only 5900 medical assistants are Saudis, which is about 27%. The report concludes that although unemployment in these sectors is high, Saudization is only 9%. Due to profit-related issues, the private sector is not keen on Saudis jobs. Such emphasis on profits is at the expense of the objectives of Vision 2030, which aims to increase economic growth, create job opportunities and invest in Saudi youth [11]. In order to address such issues under the supervision of the MOH and Social Justice, Al Nahdi Medical Company in Saudi Arabia in 2020 hired 1200 pharmacists to provide training and skill development programs. In order to compel the Private sector organizations to comply with the Saudization levels until they improve their rating, the Ministry of Labor and Social Development (MLSD) that went into effect in 26 January 2020 eliminated the yellow band of the Nitaqat program. This means that the yellow-rated companies will be automatically moved to the red band and, thus, they will not be able to apply for new visas for foreign nationals, change foreign nationals’ occupations or renew work permits until their ratings improved [12]. Furthermore, the Saudi investment minister in February 2021 emphasized that Saudization will not be forced on companies that move their headquarters to the kingdom [13].

### 3.7. Drivers of Healthcare Human Resources Supply

#### 3.7.1. Inflows

Almost all the Healthcare HR planning models reviewed are based on stock-flow approaches on the supply side in terms of the inflow of new graduates from education programs and inflow of foreign-trained health workforce.

#### 3.7.2. Education

The enrolment rates of medical students may be proportionately enhanced to meet the demand estimated. The balance needs to be supplemented with foreign medical professionals. The forecasting models for the supply of and demand for nurses usually concentrate only on the inflow of graduates and/or immigrants from other countries and on the attrition and retirement rates for graduate nurses [14]. However, semi-skilled nursing professionals can improve organizational efficiency [15] and will be needed to meet the increasing demand for care. As the demand far exceeds the supply special efforts and policy initiatives may be taken to bridge the demand-supply gap. The number of medical and nursing graduates may act as an ‘adjustment variable’.

**Figure 8:**
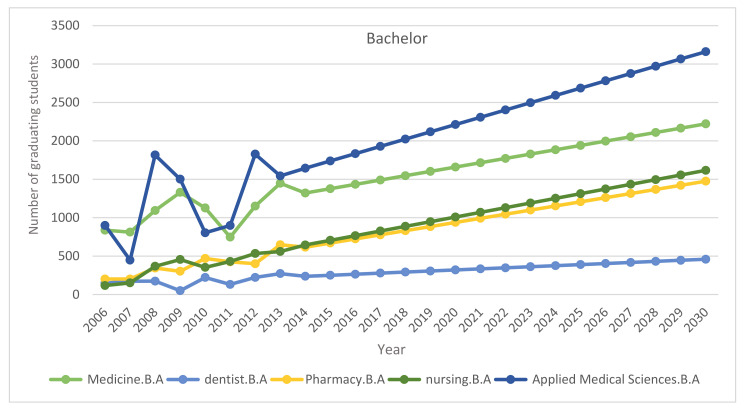
Number of graduating students from government and private medical colleges.

Figure 8 provides an estimation of the number of graduating students from government and private medical colleges in Saudi Arabia.

#### 3.7.3. Immigration

Another important inflow to the health workforce is the immigration of health workers from other countries. However, with the adoption of the 2010 WHO Global Code of Practice on the International Recruitment of Health Personnel, all countries have been encouraged to improve the planning of their health workforce requirements and refrain from recruiting health personnel from countries that are suffering from acute shortages [16].

#### 3.7.4. Outflows

We need to consider retirement as a key outflow and policy changes may be initiated to enhance the retirement age for medically fit healthcare professionals.

At the national level resignation and migration to other hospitals within the Kingdom may not affect the demand-supply gap but data on migration to other countries needs to be collected on a year-on-year basis to replenish the gap with appropriate steps whether through the enhancement of the intake of medical graduates or by encouraging medical professionals from other countries.

## 4. Policy Implications

### 4.1. Bridging the Gap between Theory and Practice

The major weakness of the nation’s healthcare policies, however, is their failure to focus on human resources [17]. Research shows that during difficult circumstances, effective workforce strategies enhance the performance of health systems [18]. Compared to other sectors, the health sector depends heavily on the availability of professionals and their competencies and effort [19,20]. Evidence shows that the human workforce drives the performance of health [21,22]. Hence, in addition to the focus on physical and technological resources, Saudi Arabia needs to invest in health human resources development and sustenance. Efforts towards organizational justice may be strengthened. Changes in technology, the use of other health workers, public expectations and government policies can all alter use patterns and the manner healthcare is delivered. There are always uncertainties regarding what new developments may occur and their impact on the demand for physician services [23].

### 4.2. Impact on Policy

Although the percentage of expatriate health professionals in the private healthcare sector is high, the results indicate that there is a positive shift towards increased health human resources in the private healthcare sector in Saudi Arabia. The implementation of the healthcare policies will enhance employment opportunities for both Saudi and non-Saudi medical professionals in Saudi Arabia and is likely to meet the healthcare needs of the growing population (Figure 7a). Furthermore, HHR policies to balance demand and supply may be initiated by the government. This can be performed by enhancing the number of seats in the medical and nursing colleges and facilities to provide opportunities to resident doctors. The demand projections (Figure 7b) indicate that there could be a shortage of Saudi doctors and nurses and, to that extent, policies to attract and retain international doctors and nurses need to be formulated in order to minimize the migration of health human resources to other countries, which is estimated to be around 20% per year. To date, physician workforce planning has not taken into account the full range of dynamic variables that are involved nor accounted for their inherent uncertainty and complex interactions [24]. The current health workforce planning literature is very much concerned with discussions and suggestions as to the optimal composition of a health workforce, but there has been remarkably little tools to assist the planner in actually determining what might be an appropriate mix of skills and personnel [25].

### 4.3. Impact on Society

The results indicate that the Saudi population is growing significantly with an average of about 49% below 29 years of age and 30% between 30–44 years of age (Figure 7a). Hence, the government needs to focus on the need for medical services across different age groups of the population and, accordingly, the demand for HHR needs to be estimated. Most of the previous HHR strategies have relied on predictive mathematical models based on the number of existing health care professionals (most often physician numbers) and changes in both total population and population demographics. However, alterations in demands related to health status (or ‘need’) or desired population health-related goals (or ‘outcomes’) were not specifically or strategically addressed. Once the relevant outcome measures are defined, the delineation of where resources are either accessible or needed must occur. Benchmarking is the most refined approach and it compares outcomes across regions by noting where they are superior and assessing what forms of health care resources are used to achieve that superior result [26]. Further studies may focus on this issue. While Saudization goal is laudable, the shortage of quality Saudi doctors and nurses needs to be bridged with international doctors and nurses with experience in the relevant specializations. There might be issues related to discrimination and, hence, efforts in this direction need to be taken to safeguard the overall healthcare requirements of the nation.

## 5. Conclusions

This study’s findings show that government policies have a significant impact on HHR in the Ministry of Health Sector, Other Government Healthcare Sector and not in the Private Healthcare Sector in Saudi Arabia. With the introduction of the 1999 law providing insurance coverage to all non-Saudis there was significant growth in the private healthcare sector. Following the Nitaqat policy in 2011 that categorized companies based on the percentage of Saudis they employed, the employment potential for Saudis increased significantly in the MOH, GOH and not in the PHS as expected. In order to enhance the employment of Saudis in the Private Healthcare Sector, which is often driven by the profit motive, the Saudi Government is taking a series of rewarding as well as punitive measures.

The results show that the number of males showing interest in nursing/allied health specialties is rising. This is a positive indication to encourage Saudization. As mentioned under Figure 1, Figure 2, Figure 3, Figure 4, Figure 5 and Figure 6, the current policies are sustainable to meet the HHR requirements in Saudi Arabia. The above results and discussion may be used as a benchmark to use entry into education programs as a measurement for future inflows from the education system. Newly trained doctors and nurses may be integrated into the projection model as they enter their professional education, upon graduation and/or upon passing a license examination or registration with a designated professional body. Dropout rates along the pathways may be analyzed to initiate remedial measures. In order to achieve the health system goals highlighted in the Kingdom’s Vision 2030, the Ministry of Health of Saudi Arabia already initiated the following strategies: development of organizational structure; administrative units; applicable rules and regulations; boosting staff efficiency; conducting research, organizational and feasibility studies needed for development; using IT advancements to automate all processes throughout MOH; enhancing information systems; as well as supervising MOH’s strategic transformation plan to automate all MOH’s facilities. The MOH recognized the importance of the human factor and its impact on MOH’s efficiency and productivity. Accordingly, the MOH started planning, organizing, guiding and monitoring talent acquisition, development, compensation and retention to achieve MOH’s objectives.

In the context of changing health care needs due to population growth, aging, chronic diseases, tight constraints on health budgets, HHR Planning and management are all likely to remain as a high priority of MOH in Saudi Arabia. The continuous development of tools and models to support policy decisions is required to meet the quantity and quality of medical and nursing education programs. The assessment of a range of possible options around health service delivery models may also be considered to meet the demand and supply gap in MOH, OGH and PHS, including the replacement of those who retire.

## Figures and Tables

**Table 1:** Healthcare professionals total inventory in 2015 and total demand for 2020 and 2030.

Sector	Category	Inventory in 2015 with a Population of 31.56 Million		Demand for 2020 with a Population of 35.09 Million		Demand for 2030 with a Population of 43.1 Million	
		Saudi (%)	Number	Saudi (%)	Number	Saudi (%)	Number
Ministry of Health	Physicians	32.6	41,240	44.3	49,456	68.0	66,555
	Nurses	60.1	95,379	71.4	117,038	92.1	160,810
	Pharmacists	91.8	3184	105.3	4840	130.4	8174
	Allied health	93.6	55,080	101.5	71,091	117.2	101,786
OGH	Physicians	50.3	16,419	49.1	19,585	48.5	27,391
	Nurses	18.2	35,119	20.9	42,559	29.9	59,503
	Pharmacists	63.6	2132	72.78	2507	88.0	3305
	Allied health	68.7	27,647	78.9	34,767	101.3	51,547
Private Sector	Physicians	2.9	29,097	−2.2	40,013	−10.7	59,516
	Nurses	5.4	41,985	9	64,681	16	105,300
	Pharmacists	3.7	18,308	8.2	28,646	16.5	47,779
	Allied health	26.6	19,046	42.6	33,486	72.9	59,532
TOTAL	Physicians	26.0	86,756	29.8	109,054	39.3	153,462
	Nurses	38.3	172,483	46.8	224,278	63.4	325,613
	Pharmacists	21.0	23,624	26.0	35,993	36.1	59,258
	Allied health	74.3	101,773	82.4	139,343	99.0	212,864

**Table 2:** Analysis of total demand and supply gap.

Sector	Category	2020				2030			
		Supply	Demand	Gap	Graduates Needed as % of Demand-Supply	Supply	Demand	Gap	Graduates Needed as % of Demand-Supply
**Total**	Physicians	9110	109,054	99,944	29.8%	28,696	153,462	124,766	39.3%
	Nurses	6148	22,4278	218,130	46.8%	22,493	325,613	303,120	63.4%
	Pharmacists	5730	35,993	30,263	26%	20,773	59,258	38,485	36.1%
	Allied health	15,244	139,343	124,099	82.4%	52,889	212,864	159,975	99.0%

## Data Availability

The data presented in this study are available on request from the corresponding author.
