# Erratum: Alnowibet et al. Healthcare Human Resources: Trends and Demand in Saudi Arabia. *Healthcare* 2021, *9*, 955

**DOI:** 10.3390/healthcare9111441

**Published:** 2021-10-26

**Authors:** Khalid Alnowibet, Adel Abduljabbar, Shafiq Ahmad, Latifah Alqasem, Nabil Alrajeh, Luigi Guiso, Mazin Zaindin, Madhusudhan Varanasi

**Affiliations:** 1 Department of Statistics and Operations Research, College of Science, King Saud University, Riyadh 11451, Saudi Arabia; lfalqasem@gmail.com (L.A.); zaindin@ksu.edu.sa (M.Z.); 2 Department of Psychology, College of Education, King Saud University, Riyadh 11451, Saudi Arabia; abduljabbar@ksu.edu.sa; 3 Department of Industrial Engineering, College of Engineering, King Saud University, Riyadh 11451, Saudi Arabia; ashafiq@ksu.edu.sa; 4 Department of Biomedical Technology, College of Applied Medical Sciences, King Saud University, Riyadh 11451, Saudi Arabia; nabil@ksu.edu.sa; 5 Department of Economics, Institute for Economics and Finance, 00118 Rome, Italy; luigi.guiso@tin.it; 6 Department of Management, College of Business Administration, Al-Yamamah University, Riyadh 11451, Saudi Arabia; v_prasad@yu.edu.sa

Some details of the authors’ names, affiliations, and email addresses were incorrect in the original version of the article [1]. Additionally, funding and acknowledgments information need to be corrected. We apologize to the readers of *Healthcare* for any inconvenience. Please see updated information as follows:

1. The correct name of the fourth author is Latifah Alqasem.
2. Affiliation number five is corrected in this form:
  ^5^ Department of Economics, Institute for Economics and Finance, 00118 Rome, Italy; luigi.guiso@tin.it
3. The correct email address of Madhusudhan Varanasi is as follows: v_prasad@yu.edu.sa.
4. The information that needs to be added to the funding and acknowledgment sections are included below.
